# Do virtual renal clinics improve access to kidney care? A preliminary impact evaluation of a virtual clinic in East London

**DOI:** 10.1186/s12882-020-1682-6

**Published:** 2020-01-10

**Authors:** S. A. Hull, V. Rajabzadeh, N. Thomas, S. Hoong, G. Dreyer, H. Rainey, N. Ashman

**Affiliations:** 1 Centre for Primary Care and Public Health, Queen Mary University of London, London E1 2AB, UK; 2 School of Health and Social Care, London South Bank University, London, UK; 3 Renal Unit, Barts Health NHS Trust, London, UK

**Keywords:** CKD, Primary care, Virtual clinic

## Abstract

**Background:**

Early identification of people with CKD in primary care, particularly those with risk factors such as diabetes and hypertension, enables proactive management and referral to specialist services for progressive disease.

The 2019 NHS Long Term Plan endorses the development of digitally-enabled services to replace the ‘unsustainable’ growth of the traditional out-patient model of care.Shared views of the complete health data available in the primary care electronic health record (EHR) can bridge the divide between primary and secondary care, and offers a practical solution to widen timely access to specialist advice.

**Methods:**

We describe an innovative community kidney service based in the renal department at Barts Health NHS Trust and four local clinical commissioning groups (CCGs) in east London. An impact evaluation of the changes in service delivery used quantitative data from the virtual CKD clinic and from the primary care electronic health records (EHR) of 166 participating practices. Survey and interview data from health professionals were used to explore changes to working practices.

**Results:**

Prior to the start of the service the general nephrology referral rate was 0.8/1000 GP registered population, this rose to 2.5/1000 registered patients by the second year of the service. The majority (> 80%) did not require a traditional outpatient appointment, but could be managed with written advice for the referring clinician. The wait for specialist advice fell from 64 to 6 days. General practitioners (GPs) had positive views of the service, valuing the rapid response to clinical questions and improved access for patients unable to travel to clinic. They also reported improved confidence in managing CKD, and high levels of patient satisfaction. Nephrologists valued seeing the entire primary care record but reported concerns about the volume of referrals and changes to working practices.

**Conclusions:**

‘Virtual’ specialist services using shared access to the complete primary care EHR are feasible and can expand capacity to deliver timely advice. To use both specialist and generalist expertise efficiently these services require support from community interventions which engage primary care clinicians in a data driven programme of service improvement.

## Background

In the adult UK population the estimated prevalence of Chronic Kidney Disease (CKD) stages 3–5 is 5–6% [1]. Identification and coding of CKD in primary care provides a case register which can be used to support the active management of blood pressure, cardiovascular risk and safer prescribing. A register also enables regular review of CKD and specialist referral where there is diagnostic uncertainty or evidence of progressive disease [2].

There is some evidence that lowering blood pressure can delay the progression of CKD [3, 4]. The high rates of cardiovascular risk associated with CKD can be reduced by advising the use of statins and improving control of blood pressure [5].

Currently almost 70% of health and social care budgets are directed towards the care of people with long term conditions [6]. The NHS Long Term Plan, released in 2019, envisages efficiencies in the management of chronic diseases and major changes to the delivery of hospital outpatient care which is described as outdated and unsustainable. It endorses digitally-enabled primary and outpatient care, which ‘*will go mainstream across the NHS’*, and ‘*will free up significant medical and nursing time’*. [7]

A number of UK studies describe a variety of virtual renal clinics which include alternatives to face to face consultations. Harnett et al. describe discharge from a general nephrology clinic into virtual follow-up, with regular test monitoring organised by the hospital and communicated to the patient’s GP [8]. Jones et al. describe a shared primary-secondary care scheme with nephrologists monitoring the test data recorded in primary care [9]. Mark et al. triaged less-complex referrals to virtual biochemical surveillance, and demonstrate the cost saving compared to routine clinic attendance [10].

Other approaches which use structured test monitoring independent of clinic attendance include eGFR graph surveillance by laboratory staff, this is specifically designed to identify those with progressive CKD and encourage onward referral [11].

In contrast with these schemes, which are run from hospital clinics, the east London community service includes dashboard data on every GP registered patient with biochemical evidence of CKD [3–5], not only those referred into renal clinics. It has an emphasis on upstream CKD management in primary care (blood pressure control and statin prescribing) with benefits for reducing the risk of cardiovascular disease associated with a declining eGFR [12]. All general nephrology referrals from GPs are assessed in the virtual clinic. The clinic aims to support the management of less-complex CKD within the framework of primary care management of long-term conditions, by providing timely advice, but restricting traditional outpatient clinic follow up to the small number of progressive cases which require more intensive specialist management.

### Aims

a) To describe the development of a virtual CKD clinic set within a community kidney service which integrates data across primary and secondary care, based on the concept of a *learning health system* – in which the data from every patient encounter is used for system development and better practice [13].

b) To evaluate the impact of the virtual CKD clinic on timely access to specialist advice, and on satisfaction with changes to service delivery by primary care clinicians and renal specialists.

## Methods

### Study design and setting

This observational study was set in east London primary care and the Renal Unit at Barts Health NHS Trust between 2015 and 2018. Barts Health NHS Trust is the sole tertiary renal provider for North-East London, reporting a high incident need for renal replacement services, with over 30% of patients with new end stage renal disease commencing dialysis in an unplanned manner, compared to 15.6% across the UK as a whole [14].

All 130 GP practices in the three contiguous inner east London clinical commissioning groups (CCGs) co-terminus with their London boroughs of City and Hackney, Newham and Tower Hamlets (total population 800,000) were involved in the first stage of the service change during 2016, with 36 practices in Waltham Forest CCG joining in 2017. All practices use Egton Medical Information Systems (EMIS Web) for the patient electronic health record. In the 2011 UK Census, almost half of the population in each of these CCGs was recorded to be of non-white ethnic origin [15], and the English indices of deprivation 2015 show that all three inner east London localities fall in the lowest decile for social deprivation in England [16].

### Theoretical stance

Many of the strategies for change management described by Kotter [17] were used in developing the design and implementation of this programme. These include: building the case for change and forming a guiding coalition which includes both clinicians and managers, empowering others to act on the programme by providing clinical information, quality improvement (QI) tools and comparative performance data, ensuring that there are early wins for the programme and building sustainability for the future by embedding the new approach into work as usual.

We are also aware of the importance of local context in determining the uptake and successful implementation of change. This project builds on previous experience of successful quality improvement projects in participating CCGs.

### Description of the East London community kidney service

The community kidney service had three components described below. This report focuses on the evaluation of the virtual CKD clinic.

1) *The virtual CKD clinic:* this takes electronic-only referrals from GPs for general nephrology advice into a weekly hospital clinic serving each CCG. Service development included the introduction of the EMIS Web platform to the renal unit and sign up by all practices to data sharing agreements to allow nephrologists to view the complete primary care electronic health record (EHR), with informed patient consent. This facilitates review of eGFR plots over time, proteinuria and all recorded investigations, examinations, medication history, co-morbidities, hospitalisations and other specialist in- and out-patient documents.

Following review of the notes, nephrologists record advice in their version of EMIS Web, which is immediately available for all clinicians in the practice to view. On average each virtual consultation took 20 min, this compares to a first attendance out-patient template time slot at Barts Health and other Renal Units of 30 min per new patient for a general nephrology consultation. GPs are advised when the nephrologist has ‘seen’ their patient by an alert within the EMIS workflow module. The clinic has a short wait time with the aim of providing timely clinical advice for GPs. Nephrologists triage the minority of patients who require further investigation into traditional, face to face, general nephrology out-patient clinics. Each CCG community clinic has 2–3 named nephrologists, with the aim of building positive clinical relationships between GPs and hospital based specialists.

Each participating CCG agreed additional pilot funding to initiate these changes to the renal service. The ambition was to fund the service as a block contract based on the previous years’ general nephrology activity. The NHS Long Term Plan increasingly commissions for whole pathways rather than itemised episodes of care. The per CCG contract for the virtual system was priced at an annually reviewable, fixed tariff for all activity, including education, developing and delivering dashboards, and practice facilitation. Hence the Renal Unit carried the risk of growth in appointments and activity above baseline, with CCGs holding the risk of the service contracting traditional outpatient activity and GPs providing more extended management in primary care settings. In addition each CCG developed customised local enhanced services (activity additional to the GP core contract [18]) with financial incentives to promote best CKD management (treating blood pressure to target, use of statins for secondary CVD prevention and monitoring CKD progression.) Quarterly dashboards identified the number of patients with evidence of CKD, changes in practice performance in CKD coding and management, and were available to practices, commissioners and the renal department.

The first virtual CKD clinic for patients in Tower Hamlets, based at the Royal London Hospital within Barts Health NHS Trust, went live in January 2016. Roll out to Newham and City and Hackney took place 6 months later, and Waltham Forest joined the programme in 2017.

The other elements of the community service included: 2) *A package of IT tools:* these enable practices to identify patients who require diagnostic coding, would benefit from better blood pressure control, or an offer of statins to decrease the risk of cardiovascular disease. They also include monthly practice alerts to identify patients with a falling estimated glomerular filtration rate (eGFR). The ‘falling eGFR trigger tool’ reports patients with an eGFR < 60 who on serial testing have a decline in MDRD-measured eGFR of ≥10 ml/min [19]. Regular practice facilitation sessions covering clinical data management and the use of project specific IT tools were provided by the Clinical Effectiveness Group (CEG) https://www.qmul.ac.uk/blizard/ceg/renal-health-service/). Extra clinical support by specialist renal nurses, with a focus on CKD management, was offered to practice teams which had the lowest rates of CKD coding.

c) *Renal education:* regular updates and case discussions for general practitioners and practice nurses were held at CCG and practice events in all three project CCGs, with specialist renal nurse-led patient education sessions for patients referred into the service [20].

### Data sources for evaluation of the virtual CKD clinic

Data on referrals, appointment numbers, cost and type (whether virtual, traditional general nephrology outpatient first or follow up attendance) and wait time were collected from the care records system (CRS) at Barts Health NHS Trust. This was supplemented by nephrology department data on transfers between virtual and traditional appointments, and on renal follow up of patients in the virtual clinics.

Anonymised data on practice coding and primary care management were collected on a quarterly basis through EMIS Web and collated into practice and CCG level dashboards.

Questionnaire survey data from Tower Hamlets GPs (the pilot locality for the virtual clinic service) was collected soon after the clinic went live and before the service had become ‘work as normal’. This data was enriched with interviews with GPs recruited from Tower Hamlets practices, and all three nephrologists involved in delivering virtual clinics. These individual interviews with seven GPs, three nephrologist and one CEG facilitator were recorded and transcribed and a thematic analysis using the Framework approach was adopted [21, 22]. Two members of the research team reviewed the text to ensure trustworthiness of the data. The thematic analysis focussed on the perceived benefits and limitations of the new service. The survey questions and interview topic guide are shown in Additional file 1: Table S1.

All data were anonymised and managed according to UK NHS information governance requirements. Ethics approval was not required for this service evaluation, as all patient-level data are anonymised, and only aggregated patient data are reported in this study.

## Results

In the four contiguous participating CCGs of Tower Hamlets, Newham, City & Hackney and Waltham Forest, with a GP registered population of 1.2 million people in 2017, there were 21,560 adults with biochemical evidence of CKD (stages 3–5) at the mid-point of the study. This population prevalence of 1.8% is similar to that of London as a whole (1.9%). The figure reflects the young London population, and probable under ascertainment of CKD. The dashboard showing variation in CKD coding rates and primary care management of CKD across the four CCGs is shown in Table 1.

**Table 1:** East London CKD dashboard January 2017: 21,560 adults with biochemical evidence of CKD (stages 3–5), from four participating CCGs

METRIC	CCG 1	CCG 2	CCG 3	CCG 4
1. Proportion of CKD cases coded	87%	80%	54%	49%
2. Proportion of CKD cases, with diabetes, coded	88%	83%	59%	60%
3. CKD with BP below 140/90	74%	71%	64%	55%
4. CKD and diabetes, with BP below 130/80	43%	39%	36%	31%
5. Adults with CKD on lipid lowering medication	80%	76%	73%	64%

The majority of practices engaged with the IT tools, and within the first year CKD coding rates improved, with the lowest coding CCG improving performance by 50% [23].

### Referrals to the virtual CKD clinic

From the start of the service all routine general nephrology referrals from GPs were processed through the virtual clinic. GPs were encouraged to refer anyone they would previously have sent to out-patients, and received local guidance which conformed to the 2014 NICE CKD guidelines [2]. The ‘falling eGFR’ trigger tool, run monthly in practices, also identified cases to be considered for referral, on average 8% of trigger tool cases were referred to the virtual clinic.

**Fig. 1:**
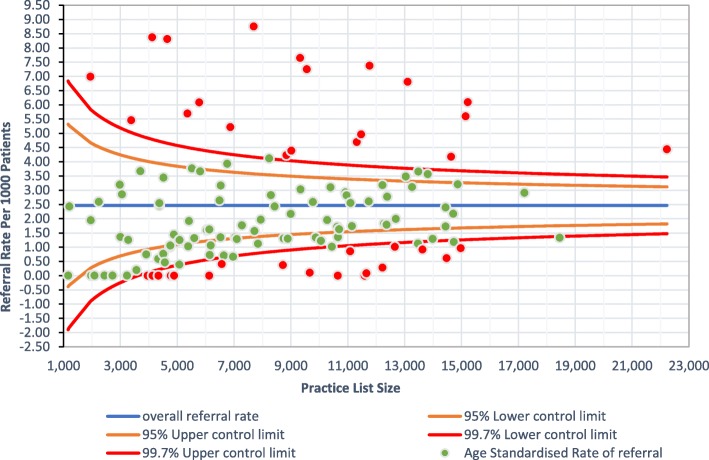
Annual (2018) age adjusted referrals to the virtual CKD clinic from 130 participating practices in east London.* *Practice populations age standardised to the East London population at the study mid-point.

In the 12 months prior to April 2015 the average annual referral rate to general nephrology outpatient clinics was 0.8/1000 GP registered population. By the second year of the service (2018) the average, annual referral rate was 2.5/1000 registered patients as shown in the funnel plot (Fig. 1). This graphic shows that 15% of practices fell outside the upper control limit for referrals, and four practices with a list size > 9000 made no referrals during the year.

### Clinic data

The average waiting time from GP referral to a first outpatient appointment in 2015 was 64 days. When the virtual clinic started the average time between GP referral and virtual clinic assessment fell to 4–6 days. The nephrology opinion can be viewed in the GP record on the day it is written, and a clinic notification is sent electronically to the practice within a few days.

**Fig. 2:**
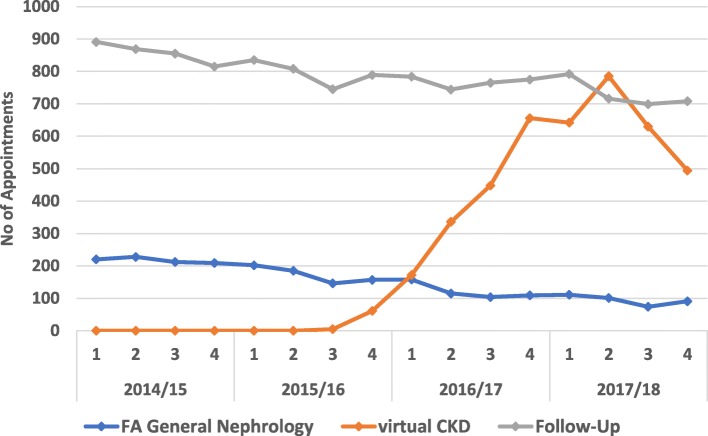
First appointments in general nephrology, numbers of virtual clinic and follow-up appointments for all participating practices in east London: quarterly 2014–18.* *Financial year quarters.

Figure 2 shows the rapid take up of the virtual clinic with an unexpected threefold rise in appointments over the first 2 years of the service for all four CCGs combined. (Additional file 1: Figure S1. shows appointment details for each of the four participating CCGs). Over the 2 years following implementation the number of first general nephrology outpatient appointments had halved, and the number of follow-up appointments showed a steady decline. These changes have released general nephrology clinic appointments to be used for closer review of specialist and more complex cases.

Across the whole service nephrologists arrange an outpatient face-to-face review following just 12% of virtual appointments. Over 40% are discharged back to the GP, with up to 50% being tagged for a further specialist review in the virtual clinic (see Table 2).

**Table 2:** Outcomes of first virtual CKD clinic appointment: 2016–18

Outcomes of first virtual CKD appointment: showing variation across the four participating CCGs	
First referrals to vCKD during 2016–18	1819
Discharge to GP	35 to 47%^a^
Face-to-face out-patient appointment	9 to 14%^a^
Review in the virtual clinic	40 to 56%^a^

^a^lowest and highest figures across the four CCGs

**Fig. 3:**
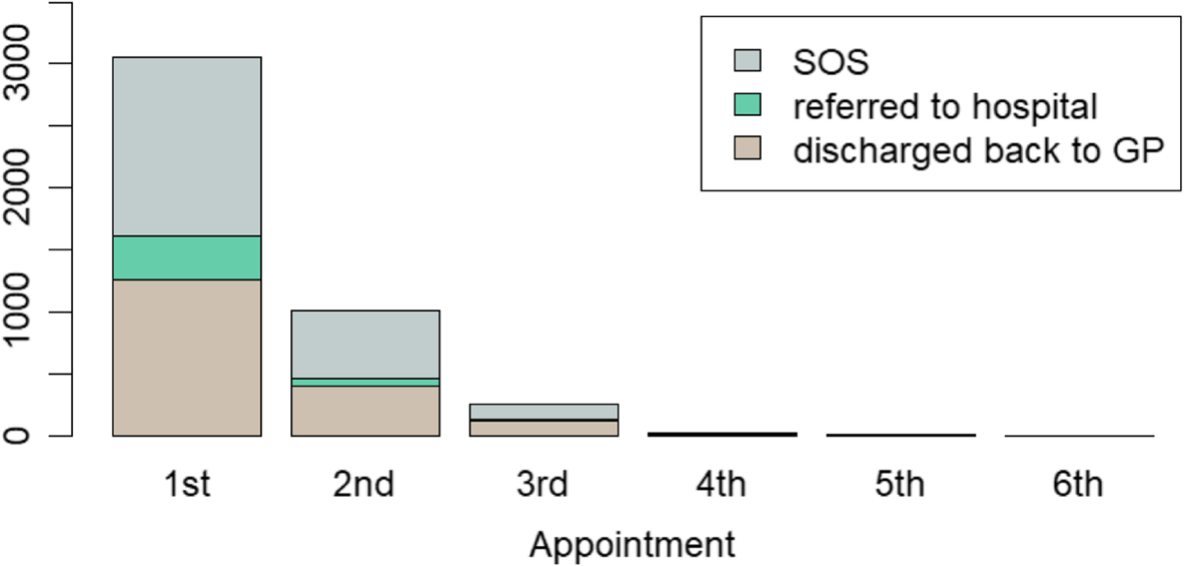
Virtual Clinic outcomes by first and follow up virtual appointment for the period April 2017 March 2018 for all four CCGs.* *The vertical axis shows the number of appointments, the horizontal axis tracks the number of virtual appointments for individual patients. SOS = review appointment in the virtual clinic.

It was also possible to measure the ‘*hidden work’* associated with virtual clinics by observing the repeated virtual reviews done by nephrologists. More than 40% of initial referrals had a second virtual review, and 30% of these had a third review (Fig. 3). The repeated review of virtual referrals was often linked to requests to GPs to arrange further investigations to facilitate a more complete assessment. This virtual review work made up approximately 50% of a virtual clinic session, and alongside the early surge in new referrals contributed to a perception of overload by nephrologists. This work was not transparently captured by routine hospital recording systems.

### Survey and interview data

**Fig. 4:**
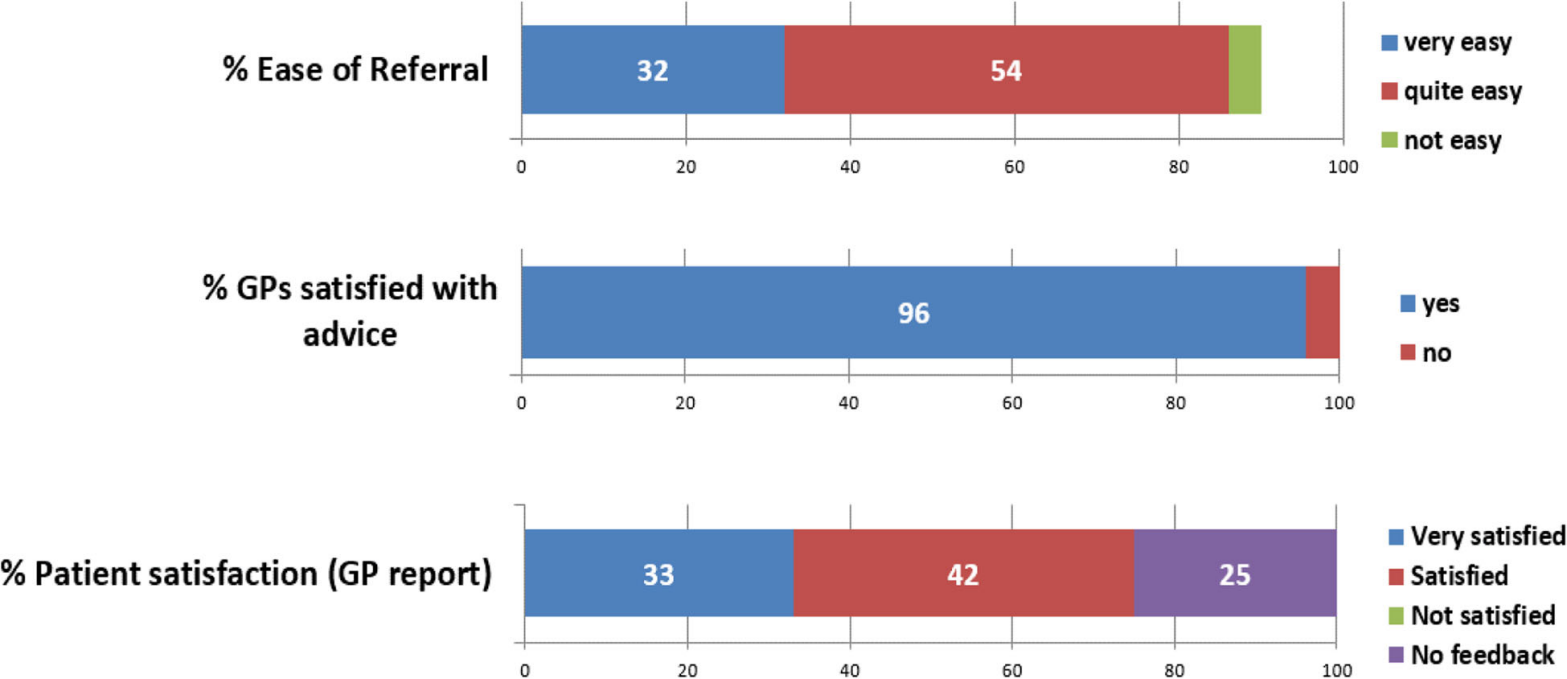
Virtual CKD clinic survey in Tower Hamlets CCG (28 responses from 68 GPs)

During the first year of the service a questionnaire was sent to all 68 GPs in Tower Hamlets who had used the virtual clinic. There were 28 (41%) responses, with 86% of responders reporting that it was very or quite easy to use the service and 96% being happy with the referral advice they received from the nephrologist (Fig. 4). GPs reported that most patients were satisfied with the service although one quarter reported no feedback from patients. The overall value of the new kidney service was rated as 5/5 by 60% of respondents.

### Key themes from the interviews

#### Benefits for patients

Every GP interviewed said that all patients had readily consented to their records being shared, with many expressing surprise that this was not already happening:

“*I think the system is great, and keeping people out of hospital is clearly a good thing.”*

Some GPs described how patients were now being referred when they had not been in the past:

“*It is useful to get a bit of advice. In the past I would probably have not done anything to be honest….as they (the patients in the nursing home) were not fit enough to go up to hospital”*

The timeliness of the referral was also considered important for patients.

*“Having the nephrologists seeing people within one week is a great benefit.”*

Finally, a number of GPs spoke about how the new system was educating them in managing CKD in the future.

*“The quality of the information coming back is good….I understand a bit more now about the tests.”*

#### Working relationships between primary and secondary care

Many GPs spoke of the improved relationship with secondary care, particularly in terms of better communication and improved continuity of care:

“*We have never met these people (the nephrologists) but I feel I have a relationship with them now….you cannot underestimate that.*”

“*The personal contact – I get the impression that there is better continuity as there is a named nephrologist.*”

Nephrologists reported that in the old system, some patients were referred but did not actually need to be seen, often there was no referral letter and up to half the time there were incomplete notes in clinic. Other challenges included the duplication of tests, not knowing the medication list, transport or language difficulties.

“*What I was not doing was anything meaningful.”*

The referral process is now easier with quicker response times. The ability to see the full record, including all tests and correspondence allows a more in-depth case-review. From the nephrologists’ perspective this can be challenging. Virtual reviews take about the same time as an outpatient slot, and for some there is a sense of regret as patient contact has diminished:

*“The workload has surprised me…. It is a lot - I spend probably the same amount of time…10 new patients in 4 hours.”*

*“You do miss the sense of the person…you don’t have the same sense as if someone is sitting in front of you.”*

There was a new respect for each other’s role. One said of the old system:

*“There was no thought in my mind that I would discharge them back to the GP with an agreed common plan.”*

but now:

“*That was a big mind shift for me, GPs and nephrologists don’t often see each other’s work of value*.”

The key messages are that patients are content to share their primary care record with nephrologists, so that management advice can be obtained without needing a visit to the hospital. The service provides timely advice back to GPs, who value the improved relationship with the nephrologists. One nephrologist concluded by saying:

*“There’s a lot of kidney disease out there that we did not know anything about.”*

## Discussion

### Main findings

Over a 3 year period this project developed a complex intervention to improve primary care management of CKD and provide timely access to specialist advice.

The introduction of this unique virtual CKD clinic, based on sharing full access to the primary care record, was followed by rapid take up of the service by local GPs across all four participating CCGs. Improved access to specialist advice also included disadvantaged groups, such as care home residents, for whom traditional OPD attendance poses most difficulties. Clinic barriers to effective assessment, such as lost notes, transport delays, language barriers and patient non-attendance simply disappeared. Time from GP referral to nephrology advice visible in the GP record fell from 64 to 6 days. Surveys identified high rates of satisfaction from GPs with ease of clinic use, the value of timely specialist advice and increased confidence in managing CKD. Nephrologists valued seeing the entire patient record, particularly the eGFR graph, but were more affected by changes to traditional working practices and the loss of patient contact. Virtual assessment took somewhat less time than a traditional first outpatient appointment. Only 12% of referrals required a subsequent face-to-face appointment, however 40–50% of referrals had at least one virtual follow up before discharge back to the GP. The service change was highly successful in providing an expansion in the capacity to assess patients with kidney disease and provided rapid access to traditional outpatient services for the small numbers who needed specialist investigation and follow up.

### Strengths and limitations

A strength of this project is that all general practices across four contiguous CCGs took part. Hence this evaluation examines the application of a complex service change to whole health economies, rather than just selected practices. The first year of the project involved three CCGs which already had a well-developed working relationship with the Clinical Effectiveness Group and the primary care data management, practice comparisons and facilitation services they offer [24]. Historically the clinicians and managers in these CCGs have been early adopters of clinical change of value to patients and the health economy. The project was slower to engage with the fourth CCG (Waltham Forest) where there was less experience of data sharing and system wide quality improvement work.

The programme evaluation was pragmatic, and allowed for some variation in the way the intervention was implemented in each of the four CCGs. Identifying contextual differences between CCGs in clinical leadership, and in their approach to incentivising change in their constituent practices is important for the success of scaling up complex interventions such as this. Differences in context can affect the process of implementation and contribute to differences in speed of diffusion across differing geographical areas.

This evaluation is limited to the impact on timely and inclusive access to kidney services. We do not have data on the clinical outcomes for patients and recognise that a longer evaluation period is needed to fully understand the impact of the change, over time and against prior practice.

### Implications for practice and future research

Changes to the traditional patterns of delivering care will involve a complex interaction between making the most effective use of data within the electronic health record (EHR) both in primary and secondary care, harnessing clinical leadership to develop novel care pathways, and utilising information technology to expand patient engagement in their healthcare. The Wachter report [25] pointed out that digitisation is only one part of a whole system of change and that: “..*implementing health IT is one of the most complex adaptive changes in the history of healthcare, and perhaps of any industry. Adaptive change involves substantial and long-lasting engagement between the leaders implementing the changes and the individuals on the front lines who are tasked with making them work.”*

Data collected for the project was used to improve the system. Examples include the referral funnel plot which is used to identify practice teams which might benefit from clinical visits, and the measurement of the ‘*hidden work’* within virtual clinics which questioned the frequency of virtual follow up by clinicians. The learning health system we describe has implications for changes to clinical practice nationwide. Interventions such as this will all require improvements to the interoperability of IT systems to deliver clinically useful real-time data for both GP practices and hospitals, and will benefit from regular facilitation to engage clinical teams in the effective use of IT for clinical quality improvement. They also require positive working relationships between managers and clinicians across primary and secondary care to enable the necessary engagement in data sharing for learning along the whole patient pathway.

## Conclusions

The kidney service described here illustrates that it is feasible to develop ‘virtual’ specialist services by sharing access to the complete primary care EHR. For such services to thrive they need support from community interventions which enlist primary care in a continuous process of service improvement, and hence can make best use of both specialist and generalist expertise. Replacing hospital attendance for CKD with specialist review of the EHR appears acceptable to patients. However, such ‘virtual’ services widen access, increase referrals, and hence do not reduce the overall clinician time needed for decision making. Further work is needed to ensure that the clinical outcomes for patients are at least commensurate with those in traditional out-patient settings. This community service has lessons for the efficient delivery of kidney services nationally. The full implications of such changes in the structure of care need further exploration before applying them to specialist clinics in other long term conditions.

## Supplementary information

**Additional file 1: Table S1.** (a) Semistructured questionnaire for interview with GPs and nephrologists. Details of questions asked during one to one interviews with clinicians(**b)** General practice survey questions. Details of 8 survey questions circulated to general practices soon after the start of the service implementation. **Figure S1.** First appointments in general nephrology, numbers of virtual clinic and follow-up appointments for each of the four CCGs participating in the community renal service: quarterly data 2014–18. A graph showing the appointment data broken down for each of the four participating CCGs in the project.

## Data Availability

The datasets generated and/or analysed during the current study are available from the corresponding author on reasonable request.

## Abbreviations

CCG: Clinical commissioning group (https://www.nhscc.org/ccgs/)
CEG: Clinical effectiveness group
CKD: Chronic kidney disease
CRS: Care records system
CVD: Cardiovascular disease
eGFR: estimated glomerular filtration rate
EHR: Electronic health record
EMIS: Egton Medical Information Systems
GP: General practitioner
IT: Information technology
NHS: National health service
OPD: Out patient department
