# Supplementary material for: Do virtual renal clinics improve access to kidney care? A preliminary impact evaluation of a virtual clinic in East London
Source: BMC Nephrol. 2020 Jan 10;21:10. doi: 10.1186/s12882-020-1682-6 (PMC6954525; doi:10.1186/s12882-020-1682-6)
Supplement: Supplementary file 1 — Additional file 1: Table S1. (a) Semistructured questionnaire for interview with GPs and nephrologists. Details of questions asked during one to one interviews with clinicians(b) General practice survey questions. Details of 8 survey questions circulated to general practices soon after the start of the service implementation. Figure S1. First appointments in general nephrology, numbers of virtual clinic and follow-up appointments for each of the four CCGs participating in the community renal service: quarterly data 2014–18. A graph showing the appointment data broken down for each of the four participating CCGs in the project. [file 12882_2020_1682_MOESM1_ESM.docx]

**ADDITIONAL FILE 1**

**a)Semistructured questionnaire for interview with GPs and nephrologists**

| Theme | Questions |
| --- | --- |
| How was it before? | Experience of referring to renal, frequency, ease, satisfaction with response, assessment of length of wait for appointment |
| How is it now? | Ease of using new referral system  Satisfaction with response speed of response transfer of work to GPs? |
| Benefits (and potential problems) | Seeing the whole record (not just what GP puts in letter) Building relationship with locality nephrologist  Work transfer to primary care?  Increased responsibility within primary care?  No follow up in nephrology – is this a risk for patients |
| How do patients see this? | Communication with patients about data sharing  Do patients miss having a F2F appointment at OPD?  How do GPs communicate back to patients after the e-clinic?  What are the risks for patients? |
| For consultants | How easy is this way of working? What are the benefits of seeing the shared record?  How should it be taken forward? |
| Looking broader | Is this a model for other departments?  Would it work for other specialties? This may impact on the FCE model of secondary care? |
|  | Could anything be done differently? |

**b) General practice survey questions**

**Evaluation of East London Community Kidney Service in Tower Hamlets (virtual CKD clinics)**

There are eight short questions and places to add free text if you wish. The survey will take 5 minutes to complete.

1. **Approximately how many referrals have you made to the community kidney service since the service began?**

- None
- Under 5
- 5-10
- More than 10
- I am not sure

**2. How easy is it to make online referrals to the community kidney service?**

- Very easy
- Quite easy
- Not easy
- If not easy please explain why

3. **On a scale from 0 to 5, where 0 is the least timely and 5 is the most timely, how timely was the response to your referral following submission?**

0 1 2 3 4 5

4. **On a scale or 1 to 5, where 0 is the least satisfied and 5 is the most satisfied, how satisfied are you with the quality of the referral advice you have received following referral of a patient to the community kidney service?**

- Yes
- No
- If no, please explain why

**5. How clear are you about what to do following referral advice from the nephrologist?**

- Very clear
- Quite clear
- Not at all clear
- If not clear, please explain why

**6. How satisfied have patients been with the new kidney service?**

- Very satisfied
- Satisfied
- Not satisfied
- I have not had feedback from patients

**7. On a scale of 0 to 5, where 5 is the most familiar, how familiar are you with the patient education aspects of the service?**

0 1 2 3 4 5

8. **On a scale of 0 to 5, where 5 is the most valuable, how would you rate the overall value of the community kidney service?**

0 1 2 3 4 5

**Please add any other comments about the service in the box below.**

**ADDITIONAL FILE 2**

**1. First appointments in general nephrology, numbers of virtual clinic and follow-up appointments for each of the four CCGs participating in the community renal service: quarterly data 2014-18.**

**Tower Hamlets**

**City & Hackney**

**Newham**

**Waltham Forest**
